# Le psoriasis du gland chez le sujet circoncis

**DOI:** 10.11604/pamj.2020.35.122.17417

**Published:** 2020-04-15

**Authors:** Soufiane Ennaciri, Moulay Hassan Farih

**Affiliations:** 1 Service d'Urologie, Centre Hospitalier Universitaire Hassan II, Fès, Maroc

**Keywords:** Gland, psoriasis, l’homme circoncis, Gland, psoriasis, circumcised man

## Abstract

Psoriasis only affecting the genital area occurs in 2-5% of subjects with psoriasis. This condition is a frequent reason for consultation with a specialist in men’s genital mucosa, since it accounts for 24% of the reasons for consultation. The gland of circumcised subjects is usually finely squamous and non-squamous in non-circumcised subjects, based on preputial moisture. Gland involvement can be diffuse and associated with an involvement of the internal face of the prepuce. We here report the case of a 30-year old patient with a family history (grandfather) of psoriasis. The patient involved in the study had a 2-month history of squamous circumferential erythematous lesions on the glans penis without pruritus or associated urinary signs and without cutaneous manifestations. Biopsy of the mucosa of the glans penis was performed which confirmed the diagnosis of psoriasis.

## Image en médecine

L'atteinte génitale exclusive du psoriasis ne concerne que 2 à 5% des psoriasiques. Cette affection constitue un motif fréquent dans les consultations spécialisées dédiées aux muqueuses génitales masculines, puisqu'elle représente jusqu'à 24% des motifs de consultation. Sur le gland, l'atteinte est habituellement finement squameuse chez le sujet circoncis et non squameuse chez le non circoncis, du fait de l'humidité favorisée par le prépuce. L'atteinte du gland peut être diffuse et s'associer à une atteinte de la face interne du prépuce. Nous présentons le cas d'un jeune patient de 30 ans, qui a comme antécédent un grand parent atteint de psoriasis. Notre patient a vu apparaitre depuis 2 mois des lésions érythémateuses squameuses circonférentielles au niveau du gland sans prurit ni signes urinaires associées et sans manifestations cutanées. Une biopsie de la muqueuse du gland a été réalisée ayant confirmé le diagnostic de psoriasis.

**Figure 1:**
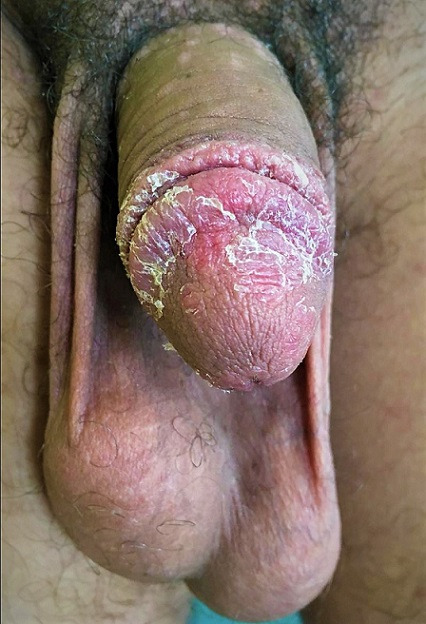
Image photographique montrant un psoriasis exclusif du gland chez un sujet circoncis, se manifestant par des lésions érythémateuses et surtout squameuses

